# E-Cigarette Use in a Country With Prevalent Tobacco Smoking: A Population-Based Study in Taiwan

**DOI:** 10.2188/jea.JE20170300

**Published:** 2019-04-05

**Authors:** Yi-Lung Chen, Shang-Chi Wu, Yen-Tyng Chen, Po-Chang Hsiao, Ya-Hui Yu, Te-Tien Ting, Chuan-Yu Chen, Yu-Kang Tu, Jiun-Hau Huang, Hao-Jan Yang, Chung-Yi Li, Carol Strong, Cheng-Fang Yen, Chia-Feng Yen, Wei J. Chen

**Affiliations:** 1 Institute of Epidemiology and Preventive Medicine, College of Public Health, National Taiwan University, Taipei, Taiwan; 2 Department of Behavioral Sciences and Health Education, Rollins School of Public Health, Emory University, Atlanta, GA, USA; 3 Genetic Epidemiology Core, Center of Genomic Medicine, National Taiwan University, Taipei, Taiwan; 4 School of Big Data Management, Soochow University, Taipei, Taiwan; 5 Department of Public Health, College of Public Health, National Taiwan University, Taipei, Taiwan; 6 Institute of Public Health, National Yang-Ming University, Taipei, Taiwan; 7 Center of Neuropsychiatric Research, National Health Research Institutes, Zhunan, Miaoli County, Taiwan; 8 Institute of Health Behaviors and Community Sciences, College of Public Health, National Taiwan University, Taipei, Taiwan; 9 Department of Public Health, College of Health Care and Management, Chung Shan Medical University, Taichung, Taiwan; 10 Department of Public Health, College of Medicine, National Cheng Kung University, Tainan, Taiwan; 11 Department of Psychiatry, Kaohsiung Medical University Hospital & School of Medicine and Graduate Institute of Medicine, College of Medicine, Kaohsiung Medical University, Kaohsiung, Taiwan; 12 Department of Public Health, College of Medicine, Tzu Chi University, Hualien, Taiwan

**Keywords:** electronic cigarettes, smoking, policy, tobacco

## Abstract

**Background:**

The different profiles of e-cigarette users in different age groups have seldom been investigated, particularly in populations facing a high prevalence of cigarette smoking. This study aims to examine the prevalence and correlates of e-cigarette use separately for adolescents and adults in nationally representative samples in Taiwan.

**Methods:**

Among 17,837 participants in the 2014 National Survey of Substance Use in Taiwan, 4445 were aged 12 to 17 years and 13,392 were aged 18 to 64 years. Individuals’ lifetime tobacco use was divided into four groups: non-use, exclusive e-cigarette use, exclusive cigarette use, and dual use. Questions on sociodemographic features, use and problematic use of tobacco, alcohol, and other drugs, and psychosocial distress, among others, were administered using a computer-assisted self-interview on tablet computers.

**Results:**

Among lifetime users of e-cigarette (2.2% for adults and 0.8% for adolescents), 4.5% for adults and 36.6% for adolescents were exclusive e-cigarette users. From use of exclusive e-cigarettes to use of exclusive cigarettes to dual use, those usage groups were related to an increasing trend of adjusted odds ratios for use of other psychoactive substances, particularly problematic use of alcohol or drugs, and with more depressive symptoms. Two correlates were specific to e-cigarette use: alcohol use had stronger relationships with e-cigarette use among adolescents, and younger adults (18–34) were more likely to try e-cigarettes compared to older adults.

**Conclusions:**

These results provide essential information regarding e-cigarette use in the general population, and future prevention strategies should account for its specific correlates in young people.

## INTRODUCTION

Cigarette smoking accounts for a notable proportion of the global burden of disease.^1^ The prevalence of tobacco smoking is especially high in South, Southeast, and East Asia.^2^ For example, Taiwan had a national lifetime prevalence of cigarette smoking of 32.1% in adults and 4.2% in adolescents in 2009.^3^ Taiwan has implemented several anti-smoking policies, including an indoor smoking ban and a tobacco surcharge. However, the declining prevalence of tobacco smoking has reached a plateau recently; the prevalence in adults was 32.5% in 1990 and 16.4% in 2013 but increased to 17.1% in 2014.^4^ Furthermore, a 3-year national survey found that the incidence rates of tobacco smoking in adolescents increased from 4.4% in 2004 to 8.4% in 2006.^5^ Preventing cigarette smoking initiation among adolescents remains a challenge for tobacco control in Taiwan.

Since the launch of electronic cigarettes (e-cigarettes) in 2005, they have been increasingly available in many countries, although they have not necessarily been legally permitted.^6^ Non-smokers’ use of e-cigarettes might increase their likelihood to smoke cigarettes.^7^ In particular, e-cigarettes with flavored e-liquids have been increasingly popular among young people.^8^ Although the importation and sales of e-cigarettes were explicitly banned in Taiwan in 2014 and have remained banned since then,^9^ there have been reports of the smuggling of e-cigarettes into Taiwan.^10^

Although there is a considerable amount of literature on the prevalence and correlates of e-cigarette use, extant studies are mainly limited to certain age groups.^11^ Many studies focused only on adults,^8^^,^^12^^–^^16^ and few studies examined adolescents^17^^–^^20^ or both adolescents and adults.^21^^–^^24^ Additionally, many empirical studies compared lifetime users of e-cigarettes with other forms of cigarette users,^18^^,^^25^^,^^26^ with few separating exclusive e-cigarette use from dual cigarettes use or dividing a common comparison group, non-e-cigarette users, into users of exclusive cigarettes and non-tobacco users. In a study that did draw a distinction between exclusive e-cigarette use and dual cigarette use in adolescents, different profiles were observed between these two groups of tobacco users.^19^ Little is known about other rarely addressed characteristics of e-cigarette users, such as substances or drugs concurrently used with e-cigarettes and the psychological well-being of e-cigarette users. Pervious study suggested a gateway role of e-cigarettes, in which use of e-cigarettes may lead to use of tobacco, alcohol, and other substances,^27^ a role that has been demonstrated in adolescents^18^^,^^19^^,^^28^ but rarely examined in adults. Given the recent availability of e-cigarettes in Taiwan, it became feasible to examine the impact of e-cigarette use on the adults of the country.

To address these research gaps and understand the impact of e-cigarettes on adolescents and adults in Taiwan, questions on the use of e-cigarettes were included in the latest National Survey of Substance Use in Taiwan in 2014. In this study, we aimed to estimate the prevalence of e-cigarette use in adolescents and adults. Furthermore, we examined the relationship of sociodemographic features, use and problematic use of other psychoactive substances, and emotional problems with e-cigarette use.

## METHODS

### Participants

The current study was based on data from the 2014 National Survey of Substance Use, which was conducted between July 2014 and December 2014 among a nationally representative sample of individuals aged 12–64 years who were non-institutionalized civilians in Taiwan, selected using stratified, multistage, probability proportional to size random sampling. The survey enrolled 17,837 participants, 4,445 of whom were adolescents (aged 12–17 years) and 13,392 of whom were adults (aged 18–64 years), with a response rate of 62.2%. Written informed consent was obtained from all participants. Detailed information, including the background, study size determination, sampling method, and study design for this study has been reported elsewhere.^29^ This study was approved by the Research Ethics Committee of the National Taiwan University Hospital (approval number: 201309034RINB).

### Measurements

Participants were asked to complete anonymously a computer-assisted self-interview on tablet computers at their homes, containing question items on sociodemographic variables, substance use, problematic substance use, and depression symptoms, among others.^29^

#### Use of conventional cigarettes and e-cigarettes

The items regarding lifetime use of cigarettes were “*Have you ever used tobacco cigarettes or cigars?*”, and “*Have you ever used electronic cigarettes?*” If the participant’s response was “Yes”, further questions regarding initiation year, cartridge consumption, and last time of use of cigarettes or e-cigarettes were asked. Past-year use was determined on whether a respondent’s last time use was within the past 1 year. Unlike the prevalence of tobacco use reported in the previous study,^29^ we adopted a different grouping strategy by separating individuals’ lifetime tobacco use into exclusive cigarette use, exclusive e-cigarette use, and dual use.

#### Sociodemographic features

Sociodemographic features were examined in the adult sample by inquiring about their gender, age, marital status, education level, and categories of occupation. Different basic information, including gender, age, truancy experience, single parent family or not, monthly allowance from their family, and employment experience, was collected in the adolescent sample.

#### Use of other substances and substance abuse

Other substances, including alcohol, betel nut, sedatives/hypnotic drugs, analgesics, and illicit drugs were also asked about. Participants who reported use of any illicit drugs, sedatives/hypnotic drugs, or analgesics were further given the 20-item Drug Abuse Screening Test (DAST) to identify individuals who were abusing psychoactive drugs.^30^

Alcohol use problems were examined using the Alcohol Use Disorders Identification Test (AUDIT).^31^ Three groups were created according to alcohol use and AUDIT score: (1) no alcohol use; (2) no alcohol use problems with a score between 0 and 7; (3) harmful alcohol use with a score of 8 or greater.^32^ The degree of nicotine dependence was assessed using the 6-item Fagerstrom Test for Nicotine Dependence (FTND)^33^ for participants who reported lifetime use of cigarettes.

#### Depression symptoms

Depression symptoms were assessed using the Chinese version 20-item short form Center for Epidemiological Studies Depression Scale (CES-D).^34^ Two groups were created according to the value of CES-D score: scores of 0–28 were considered a low depression score, and scores of 29–60 were considered a medium/high depression score.^35^

### Data analysis

Statistical analyses were conducted using SAS 9.4 (SAS Institute Inc., Cary, NC, USA). Prevalence data were analyzed with PROC SURVEYFREQ to deal with the complex survey design, including the Taylor-series linearization^36^ to calculate the standard error of the estimated prevalence and odds ratios.

As a preliminary analysis in comparing the slope of new e-cigarette users over different periods, the difference of two slopes was divided by the square root of the sum of the two squared standard errors.^37^ Individuals’ lifetime tobacco use was divided into four groups: (1) non-use (ie, never smoking cigarettes and never using e-cigarettes); (2) exclusive cigarette use; (3) exclusive e-cigarette use; and (4) dual use.

Group comparisons were conducted using the chi-square test (for categorical variables) or *t* test (for continuous variables). We conducted multivariable multinomial logistic regression analyses of tobacco usage groups on variables of interest with other sociodemographic features as covariates to derive the adjusted odds ratio (aOR) and its 95% confidence interval (CI), which represents the magnitude of association with a specific tobacco usage group after adjustment for the sociodemographic features included in the model. Furthermore, a proportional odds logistic regression analysis was conducted to test for trends in the proportional odds, as compared to non-users, from exclusive e-cigarette use to exclusive cigarette use and to dual use.

## RESULTS

The distributions of demographic characteristics of the 17,837 participants in both the adolescent and adult samples were similar to the counterparts of the whole population of the nation (Table 1).

**Table 1.** Demographic characteristics of adolescent (12–17 years) adult (18–64 years) participants in 2014 National Survey on Substance Use as compared to that of the general population in Taiwan

Demographic variables	2014 National Survey			Taiwan population in 2013^a^ %

	*N*	Unweighted % (SE)	Weighted %_wt_ (SE)	
*Adolescents* (*N* = 4,445)				
Sex				
Male	2,306	51.88 (0.75)	52.08 (1.07)	52.09
Female	2,139	48.12 (0.75)	47.92 (1.07)	47.91
Age, years				
12–15	2,726	61.33 (0.74)	62.36 (1.03)	63.51
16–17	1,719	38.67 (0.74)	37.64 (1.03)	36.49
*Adults* (*N* = 13,392)				
Sex				
Male	6,616	49.40 (0.43)	49.90 (0.59)	49.96
Female	6,776	50.60 (0.43)	50.10 (0.59)	50.04
Age, years				
18–24	1,830	13.66 (0.30)	13.07 (0.38)	13.71
25–34	2,489	18.59 (0.34)	23.06 (0.54)	22.39
35–44	2,917	21.78 (0.36)	22.72 (0.50)	22.75
45**–**54	3,307	24.69 (0.37)	22.67 (0.48)	22.69
55**–**64	2,849	21.27 (0.35)	18.49 (0.43)	18.46

SE, standard error.

^a^Source: Department of Statistics, Ministry of the Interior.

### Prevalence of tobacco products usage

Table 2 displays the past-year and lifetime use prevalence for different groups of tobacco products usage. In terms of e-cigarette use, combining exclusive use with dual use, the past-year and lifetime use prevalence (0.9% and 2.0%) were much lower than that of conventional cigarettes (19.6% and 27.8%). The majority of people who used e-cigarettes were dual users, whereas only a small proportion was exclusive e-cigarette users. For example, the proportion of lifetime exclusive e-cigarette users was 4.5% (12 out of 264) for adults and 36.6% (15 out of 41) for adolescents.

**Table 2.** Prevalence of past-year and lifetime use of tobacco products in the 2014 National Survey of Substance Use in Taiwan

Tobacco product use	All (12**–**64 years) (*n* = 17,837)						Adults (18**–**64 years) (*n* = 13,392)						Adolescents (12**–**17 years) (*n* = 4,445)					
		
	Past-year use			Lifetime use			Past-year use			Lifetime use			Past-year use			Lifetime use		
					
	*N*	%_w_	(SE)^a^	*N*	%_w_	(SE)^a^	*N*	%_w_	(SE)^a^	*N*	%_w_	(SE)^a^	*N*	%_w_	(SE)^a^	*N*	%_w_	(SE)^a^
Exclusive cigarettes	2,884	18.8	(0.4)	3,916	25.9	(0.5)	2,750	20.5	(0.3)	3,700	28.2	(0.5)	134	3.2	(0.4)	216	4.7	(0.5)
E-cigarettes	137	0.9	(0.1)	305	2.0	(0.2)	126	1.0	(0.1)	264	2.2	(0.2)	21	0.5	(0.1)	41	0.8	(0.2)
Exclusive e-cigarettes	20	0.1	(0.02)	27	0.1	(0.1)	12	0.1	(0.03)	12	0.1	(0.1)	8	0.2	(0.1)	15	0.2	(0.1)
Dual use	117	0.8	(0.1)	278	1.9	(0.2)	104	0.9	(0.1)	252	2.1	(0.2)	13	0.3	(0.1)	26	0.6	(0.2)

*N*, unweighted number; %_w_, weighted prevalence; SE, Standard error; SD, Standard deviation.

^a^Estimated percentages were derived using design-based analysis of complex survey data with frequency weighting and information about stratum and primary sampling units, and variances were estimated using the Taylor series linearization method.

### Abstinence from e-cigarette use

Of note, among those who initiated use of e-cigarettes more than one year ago (*n* = 248, 22 from the exclusive e-cigarette use group and 226 from the dual-use group), 64.5% (*n* = 160, 15 from the exclusive e-cigarette use group and 145 from the dual-use group) reported no use of e-cigarettes in the past 1 year. For comparison, among those who initiated use of conventional cigarettes more than 1 year ago (*n* = 3,989), only 28.6% (*n* = 1,142) reported no use of conventional cigarettes in the past 1 year. Among 145 individuals who were dual users but stopped using e-cigarettes in the past 1 year, only 17.2% (*n* = 25) reported no use of conventional cigarettes in the past 1 year as well.

### Initiation year of e-cigarette use

**Figure 1.**
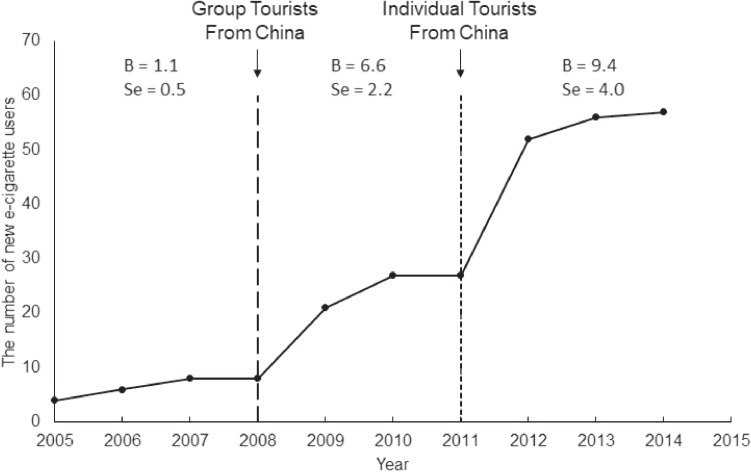
The distribution of the new e-cigarette users (*N* = 265) in the 2014 National Survey of Substance Use in Taiwan. Individuals whose self-reported initiation of e-cigarettes occurred before 2005, the year of initial introduction of e-cigarettes into the market, were not included in this analysis (*n* = 40). Three separate regression analyses were conducted for the period of 2005–2008, 2008–2011, and 2011–2014. Comparing the regression coefficients (B) of adjacent time periods, with the difference divided by the square root of the sum of the two squared standard errors (se), there was an increase from 2005–2008 to 2008–2011 (Z = 2.43; *P* = 0.008), and a tendency of increase from 2008–2011 to 2011–2014 (Z = 0.61; *P* = 0.27).

The number of new e-cigarette users continued to increase from 2005 through 2014 in the whole sample, with two sharp increases in 2008 and 2011 (Figure 1). Comparing the slopes of the three time periods demarcated by these 2 years, there was an increase from the period of 2005–2008 to the period of 2008–2011, and a tendency of increase from the period of 2008–2011 to the period of 2011–2014.

### Tobacco use and sociodemographic features

Using non-users as the reference group, the results of the multivariable multinomial logistic regression analyses are displayed in Table 3. We found that within each age stratum, tobacco smokers shared some common sociodemographic features. However, there was a trend of increasing aORs from exclusive e-cigarette use to exclusive conventional cigarette use and to dual use, which was further assessed by a trend test using proportional odds logistic regression analyses. For adolescents, this trend test of increasing proportional ORs with different tobacco usage groups was shown for individuals of male sex and older age (≥15 years old) that had a part-time job, a higher allowance (>1,000 NT dollars), came from one-parent family, and had truancy. For adults, the trend of increasing proportional ORs with different types of tobacco use was shown for individuals of male sex and who were married or divorced/widowed, had lower educational attainment, and were in the “service and sales workers” occupation group. Nevertheless, the relationships between young adulthood and the four groups of tobacco use seemed to be more J-shaped if individual aORs were examined, in which case the users of exclusive conventional cigarettes group was smallest among people aged 18–34 years compared with the other two e-cigarette use groups.

**Table 3.** Multivariable multinomial logistic regression of tobacco usage groups, using people with non-use as the reference group, on sociodemographic features in the 2014 National Survey of Substance Use in Taiwan

Variable	Non-use	Exclusive e-cigarette use		Exclusive cigarette use		Dual use		Trend test^a^
				
	*n* (%_wt_)	*n* (%_wt_)	aOR (95% CI)	*n* (%_wt_)	aOR (95% CI)	*n* (%_wt_)	aOR (95% CI)	aOR (95% CI)
*Adolescents*	(*N* = 4,188)	(*N* = 15)		(*N* = 216)		(*N* = 26)		
Male Sex	2106 (50.4)	9 (69.6)	2.4 (0.8–7.8)	170 (79.0)	4.6 (2.8–7.7)	21 (90.6)	14.7 (4.1–52.7)	4.9 (3.1–7.8)
Age, ≥15 years	2291 (54.7)	9 (70.8)	1.9 (0.6–6.5)	176 (80.8)	2.4 (1.5–3.9)	22 (90.6)	3.6 (1.0–12.5)	2.4 (1.5–3.7)
Having a job	331 (8.4)	2 (20.3)	1.9 (0.4–9.6)	71 (30.3)	3.1 (2.0–4.8)	14 (58.4)	10.1 (3.3–31.2)	3.5 (1.7–3.8)
Monthly allowance (NTD >1,000)^c^	849 (21.8)	3 (20.0)	0.7 (0.2–3.1)	77 (36.2)	1.5 (1.0–2.3)	11 (49.2)	2.2 (0.8–6.3)	1.6 (2.3–5.3)
One-parent family	661 (15.8)	4 (21.5)	1.3 (0.3–5.3)	77 (39.7)	2.8 (1.8–4.2)	10 (36.2)	1.9 (0.7–5.3)	2.6 (1.1–2.3)
Truancy	227 (5.9)	5 (31.7)	6.4 (0.8–53.2)	96 (51.6)	13.2 (6.5–26.7)	16 (62.6)	29.4 (9.8–88.2)	13.4 (7.3–24.6)
*Adults*	(*N* = 9,428)	(*N* = 12)		(*N* = 3,700)		(*N* = 252)		
Male Sex	3201 (34.3)	7 (80.6)	8.3 (1.9–35.6)	3190 (85.64)	13.4 (11.5–15.6)	218 (84.7)	11.4 (6.9–19.0)	12.7 (11.0–14.8)
Age, 18**–**34 vs 35**–**64 years	3242 (39.0)	7 (56.9)	0.6 (0.1–2.6)	954 (27.94)	1.3 (1.0–1.5)	116 (51.2)	0.5 (0.3–0.7)	1.1 (0.9–1.3)
Marital status (ref: Single)								
Married	5477 (56.1)	5 (43.1)	1.1 (0.2–5.5)	2251 (60.5)	0.8 (0.6–0.9)	123 (46.6)	0.9 (0.6–1.3)	1.3 (1.1–1.5)
Divorced or widowed	699 (6.4)	0 (0.0)	—	381 (9.6)	2.0 (1.6–2.4)	23 (8.2)	2.2 (1.2–4.1)	2.4 (1.9–3.1)
Education (ref: College or above)								
Senior high school	2,804 (27.8)	4 (48.4)	1.0 (0.1–21.3)	1,488 (39.8)	2.0 (1.7–2.4)	118 (45.2)	2.7 (1.6–4.6)	2.0 (1.7–2.4)
Junior high school or below	4,504 (52.9)	6 (43.3)	3.2 (0.3–39.1)	1,194 (36.2)	2.1 (1.9–2.5)	87 (36.1)	2.9 (2.0–4.4)	2.2 (1.9–2.5)
Occupation^b^ (ref: Group I)								
Group II	1,999 (21.1)	3 (15.8)	0.9 (0.1–8.7)	812 (23.2)	1.3 (1.1–1.6)	78 (31.5)	1.6 (1.1–2.4)	1.3 (1.2–1.6)
Group III	584 (6.0)	0 (0.0)	—	471 (12.9)	1.5 (1.2–1.8)	23 (9.2)	0.9 (0.5–1.6)	1.3 (1.1–1.6)
Group IV	3296 (33.3)	6 (12.1)	1.6 (0.1–22.4)	713 (18.6)	0.7 (0.6–0.8)	36 (11.4)	0.4 (0.2–0.6)	0.7 (0.6–0.8)

%_w_, weighted prevalence; aOR, adjusted odds ratio, with adjustment for all the listed variables in this table, either in multivariable multinomial or proportional odds logistic regression analysis.

^a^Based on proportional odds logistic regression analyses, in which the odds ratios have a linear trend going from exclusive e-cigarette use to exclusive cigarette use to dual use, using non-use as the reference group, with adjustment for the variables listed in this table separately for adolescents and adults.

^b^Occupation: group I, other occupations; group II, service and sales workers; group III, plant and machine operators and assemblers, elementary laborers; and group IV, unemployed or retired.

^c^New Taiwan Dollars (1 USD ≅ 30 NTD).

### Tobacco use and other substance use/depression

Similar analyses were conducted to determine the relationships between different tobacco usage groups and the use of other substances, problematic use of alcohol or drugs, and CES-D scores after adjustment for the associated demographic characteristics (Table 4). However, only legal substances (alcohol and betel nut), AUDIT, and CES-D scores were examined in adolescents, because use of illicit drugs was rare in the adolescent group.

**Table 4.** Multivariable multinomial logistic regression of tobacco usage groups, using people with non-use as the reference group, on other substance use and depression in the 2014 National Survey of Substance Use in Taiwan

Variable	Non-use	Exclusive e-cigarette use		Exclusive cigarette use		Dual use		Trend test^a^
				
	*n* (%_wt_)	*n* (%_wt_)	aOR (95% CI)	*n* (%_wt_)	aOR (95% CI)	*n* (%_wt_)	aOR (95% CI)	aOR (95% CI)
*Adolescents*	(*N* = 4,188)	(*N* = 15)		(*N* = 216)		(*N* = 26)		
Alcohol	1,047 (26.9)	13 (89.0)	17.1 (4.2–90.8)	161 (75.2)	4.5 (3.2–8.2)	21 (82.2)	6.0 (2.0–22.8)	5.4 (3.5–8.4)
Betel nut	58 (1.9)	2 (14.8)	7.8 (2.2–38.1)	44 (23.6)	8.3 (3.6–23.9)	13 (44.7)	18.1 (5.5–58.6)	8.6 (4.0–18.5)
AUDIT score (ref: never-user)								
Low (0–7)	1,032 (26.7)	12 (85.7)	20.1 (4.1–89.8)	143 (62.9)	5.8 (2.8–7.3)	15 (59.9)	6.9 (1.4–18.2)	4.7 (3.0–7.4)
Medium/High (≥8)	12 (0.2)	1 (3.8)	84.1 (3.9–949.9)	18 (12.3)	119.4 (19.7–168.1)	9 (22.2)	259.5 (19.3–603.2)	38.3 (17.8–82.3)
CES-D stratum (ref: Low, 0–28)								
Medium/High (29–60)	245 (5.3)	2 (13.6)	2.2 (0.5–12.3)	28 (14.86)	2.2 (1.1–5.0)	3 (21.47)	3.1 (1.1–15.5)	2.5 (1.3–4.8)
*Adults*	(*N* = 9,428)	(*N* = 12)		(*N* = 3,700)		(*N* = 252)		
Alcohol	4,562 (52.9)	7 (76.6)	3.0 (0.5–15.7)	2,978 (82.3)	4.3 (3.8–5.1)	230 (89.3)	7.3 (4.4–12.5)	4.0 (3.8–5.0)
Betel nut	381 (3.4)	2 (9.3)	1.7 (0.3–11.7)	1,727 (43.9)	9.6 (8.2–11.7)	150 (62.8)	26.1 (18.3–40.3)	9.0 (7.7–10.6)
Sedatives/Hypnotics	835 (8.9)	1 (10.1)	1.8 (0.2–15.8)	505 (14.9)	2.6 (2.0–3.1)	49 (22.5)	5.2 (3.2–8.1)	2.6 (2.1–3.1)
Non-medical use	92 (0.9)	0 (0.0)	—	93 (3.2)	4.7 (2.9–7.8)	13 (6.1)	9.3 (4.3–22.0)	4.3 (4.3–6.3)
Analgesics	942 (10.5)	1 (2.9)	0.3 (1.6–2.3)	568 (16.1)	1.9 (2.2–5.0)	62 (24.1)	3.2 (1.6–2.3)	1.9 (1.6–2.3)
Non-medical use	389 (4.2)	1 (2.9)	0.6 (0.1–7.9)	257 (7.3)	1.5 (1.6–2.7)	25 (9.9)	2.0 (1.7–5.2)	2.1 (1.6–2.6)
Illicit drug	23 (0.3)	0 (0.0)	—	108 (3.7)	16.3 (8.6–32.5)	17 (7.4)	27.1 (11.4–79.0)	7.1 (4.9–10.3)
Hard drug	7 (0.1)	0 (0.0)	—	69 (2.3)	20.8 (9.3–55.6)	9 (3.3)	27.2 (9.7–120.4)	5.3 (3.6–7.7)
Club drug only	14 (0.2)	0 (0.0)	—	35 (1.3)	15.4 (5.8–40.6)	7 (3.2)	25.3 (5.6–113.7)	10.2 (5.3–19.7)
AUDIT score (ref: never-user)								
Low (0–7)	4,386 (46.5)	5 (41.7)	1.2 (0.2–6.8)	2,212 (59.8)	3.6 (3.11–4.15)	135 (53.8)	4.7 (2.7–8.0)	3.5 (3.1–4.0)
Medium/High (≥8)	176 (2.1)	2 (16.7)	38.9 (5.1–294.8)	766 (20.7)	18.0 (13.5–24.0)	85 (33.7)	51.4 (27.6–95.5)	14.2 (11.3–17.8)
DAST score (ref: never-user/0 score)								
1–20	514 (5.3)	0 (0.0)	—	368 (10.9)	2.8 (2.3–3.7)	39 (19.3)	6.1 (4.0–11.2)	3.0 (2.4–3.7)
CES-D stratum (ref: Low, 0–28)								
Medium/High (29–60)	597 (6.9)	1 (2.0)	0.3 (0.04–2.7)	301 (8.2)	1.6 (1.3–2.2)	27 (12.4)	2.3 (1.4–4.2)	1.8 (1.4–2.3)

%_w_, weighted prevalence; aOR, adjusted odds ratio, with adjustment for all the listed variables in Table 3, either in multivariable multinomial or proportional odds logistic regression analysis; AUDIT, alcohol use disorders identification test; CES-D, center for epidemiological studies depression scale; DAST, drug abuse screening test.

^a^Based on proportional odds logistic regression analyses, in which the odds ratios have a linear trend going from exclusive e-cigarette use to exclusive cigarette use to dual use, using non-use as the reference group, with adjustment for the variables listed in this table separately for adolescents and adults.

For adolescents, the trend of increasing proportional ORs with different types of tobacco use was shown for users of alcohol or betel nut, problematic users of alcohol (via the AUDIT score), and those with depression symptoms (via the CES-D score). Nevertheless, the relationships between alcohol use and the four groups of tobacco use seemed to be more J-shaped if individual aORs were examined, in which case the users of exclusive cigarettes group had the lowest prevalence of alcohol use compared with the other two e-cigarette use groups.

For adults, the trend of increasing proportional ORs with different types of tobacco use was consistently related with use of other substances or drugs (alcohol, betel nut, sedatives/hypnotics, analgesics, and illicit drugs), nonmedical use of prescription drugs (sedatives/hypnotics and analgesics), problematic use of alcohol (via AUDIT) and drugs (via DAST), and depression (via CES-D).

**Figure 2.**
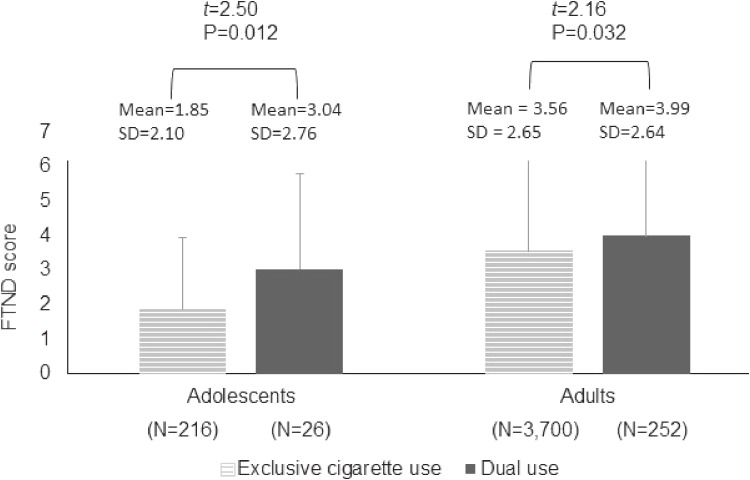
Comparing the distribution of the score on the 6-item Fagerstrom Test for Nicotine Dependence (FTND) between people with exclusive cigarette use and those with dual use in adolescents and adults, respectively.

To explore further the characteristics of dual users, we examined their cigarette use via the scores on FTND, which was inquired only for people who ever smoked cigarettes, and their e-cigarette use via the consumption amount of cartridges. Compared to exclusive cigarette users, dual users had greater degree of nicotine dependence in both adolescents and adults (Figure 2). In terms of lifetime consumption of cartridges (eg, proportion of >1 cartridge), the difference between the two groups (34.5% for dual users and 18.5% for exclusive e-cigarette users) failed to reach statistical significance.

## DISCUSSION

This study provided the empirical estimates of the prevalence of e-cigarette use in the general population in Taiwan. Despite the government’s importation ban of e-cigarettes, 2% of the population (2.2% of adults and 0.8% of adolescents) had ever used e-cigarettes, with the majority of these users having already had tobacco use experiences. We found that use of e-cigarettes alone or in combination with cigarettes was related with younger age, more use of other psychoactive substances, problematic use of alcohol or drugs, and more depressive symptoms, with an elevated risk observed that increased from users of exclusive e-cigarettes to users of exclusive conventional cigarettes to dual users. These results provided essential information regarding e-cigarette use in the general population that is critically needed for policies controlling e-cigarette use in Taiwan.

### The prevalence and trends of e-cigarette use

The national lifetime prevalence of e-cigarette use in Taiwan found in this study was similar to that of a 2015 telephone-based national surveillance in Taiwan (2.7% for the population 15 and older),^24^ but substantially lower than that of other countries (eg, 29% for adolescents in Hawaii^19^ and 12.6% for adults in the United States,^38^ 20.9% for young adults in Poland,^22^ 18.6% for the population 16 and older in the United Kingdom,^39^ 11.6% for the population 15 and older in the European Union,^40^ and 10% for adolescents in Mexico^26^). Nevertheless, our findings were closer to the estimates for Japan: 6.6% for the population 15–64 years,^41^ where e-cigarettes became legal since 2013,^41^ whereas most Western countries did so since 2006–2007.^6^ However, an e-cigarette ban itself might not effectively prevent people from accessing e-cigarettes, as the experience of Mexico youth has shown, probably due to the low risks of law enforcement and ambiguities in law.^26^ The geographic isolation of Taiwan, an island, might help stem the smuggling of e-cigarettes into Taiwan.

E-cigarette users’ self-reported initiation years revealed that the initiation of e-cigarette smoking in our sample had two time points with rapid rise in 2008 and 2011, respectively. One possible explanation is that e-cigarettes became more accessible globally, including Taiwan, after e-cigarettes entered the European market in 2006 and the United States market in 2007.^6^ Furthermore, the two surging years might be related to the loosening in cross-strait travel regulation between China and Taiwan; mainland tourists were allowed to travel as groups to Taiwan in 2008, and restrictions were further loosened in 2011 to allow mainland tourists from certain Chinese cities to travel as individuals. Since e-cigarettes have been legal since 2005 in China, and Taiwan is in the ocean area immediately north of the South China Sea,^6^ such loosening of the cross-strait travel regulations might contribute to a notable increase in the carrying-in or smuggling of e-cigarettes. These possible explanations indicate the challenge in regulating e-cigarettes in the current climate of globalization that leads to increasing traffic of people and products.

### Stability of e-cigarette use

It is intriguing that users of conventional cigarettes had a lower rate of 1-year abstinence than users of e-cigarettes (28.6% vs 64.5%). The relatively high stopping rate among e-cigarette users is consistent with previous findings.^40^ Several reasons might account for this. First, many e-cigarette users might simply have tried e-cigarettes out of curiosity. Second, the efficacy of nicotine delivery from e-cigarettes was lower than that from conventional cigarettes.^42^ This might be especially true for heavy cigarette smokers, who usually constitute the majority of e-cigarette users.^12^^,^^15^ Third, despite the advantage of being less expensive that is purported by e-cigarettes producers, the total cost of smoking e-cigarettes might actually be higher than that for conventional cigarettes.^43^

### Increasing strength of correlations from e-cigarette users to dual users

When sociodemographic correlates were examined separately for adolescents and adults, it was unsurprisingly observed that both e-cigarette users and conventional cigarette users had similar characteristics, such as sex, age group, and education level, which is consistent with previous findings.^13^^,^^23^

E-cigarette use among adolescents was also related with being from a one-parent family, having a job, and getting a monthly allowance of NTD >1,000, and these relationships have been seldom reported in the literature to date.^25^^,^^44^^–^^46^ These factors indicate that less guidance (eg, being from a one-parent family) or more financial resources might contribute to adolescent use of e-cigarettes.

On the other hand, the sociodemographic features related with adult e-cigarette use, including male gender, young adulthood, being married, and having less education, are similar to those found in previous studies.^7^^,^^21^^–^^23^^,^^47^ In addition to these features, this study further found that people with an occupation in the service and sales industry had an increased risk of using e-cigarettes. A possible explanation for this might be that the use of e-cigarettes among these individuals is a response to workplace-related stress, which has been reported to be higher among service and sales workers compared to those in other occupations.^48^

Our findings also revealed an increased risk of concurrent use of other substances and a greater magnitude of behavioral or emotional symptomatologies among the three tobacco use groups in both adolescents and adults. These findings imply that, regardless of the several purported advantages of e-cigarettes, these products contain nicotine, which has strong addictive properties, and all use groups showed similar relationships with psychosocial factors. Hence, previous explanations for the correlation between cigarette use and these psychosocial factors might be applicable to e-cigarette users as well, such as a common underlying diathesis for additive substances,^49^ augmenting effects via combined use of substances,^50^ and self-medication for depression or coping with unpleasant mood states induced by nicotine use.^51^

In both adolescents and adults in this study, there was an increasing trend in the strength of those demographic and sociobehavioral correlates among the tobacco use groups from users of exclusive e-cigarettes to users of exclusive conventional cigarettes, and to dual users, which supported the gateway of e-cigarettes to the other substance use. Although these findings have also been found in previous studies, they were limited to adolescent samples^19^^,^^52^ in Western countries. The reason why it is rarely examined in the adults may be that most adults who would use tobacco products had already done so, such that people with exclusive e-cigarette use were much fewer in adults (4.5%) than in adolescents (36.6%). A larger sample size of this particular use group is warranted to further confirm this in the future.

This trend may also be accounted for by the fact that the dual users group in this study consisted mainly of heavy cigarette users, as reflected by the greater FTND scores in the dual users group than in the users of exclusive conventional cigarettes and e-cigarettes groups. These features are consistent with previous studies reporting that dual users were usually heavy smokers.^8^^,^^12^

Two correlates seem to be specific to e-cigarette use. First, among adolescents, alcohol use seems to have stronger relationships with e-cigarette use in both users of exclusive e-cigarettes and dual users. One possible explanation is that adolescents might use e-cigarettes in settings that also provide alcohol, such as bars, pubs, and parties. Second, among adults, it is interesting to note that younger adults (18–34 years) were more likely to try e-cigarettes than older adults were, which has been reported.^26^^,^^53^ Hence, individuals designing prevention strategies for e-cigarette use should be aware of these differing correlate features of sub-populations and their related environments.

### Policy implications and future directions

Several features of e-cigarette use revealed in this study have some implications for the national policy on the regulation of tobacco products. First, despite the relatively low prevalence of past-year e-cigarette use, the increasing trend in the number of new e-cigarette users over the past decade predicts a fast increase in the future. This is contrary to the declining trend in the national prevalence of conventional cigarette use during the same period.

Furthermore, the strong relationship of e-cigarette use with the young population will render the control of tobacco use in Taiwan more difficult, given that the use prevalence of tobacco continued to increase in adolescents despite a steady decrease in adults,^4^^,^^5^ because the adolescents with use of e-cigarette has been related to increasing risk for initiation of cigarette smoking.^18^^,^^28^ Hence, this study indicates that the young population should be the primary target of efforts to prevent e-cigarette use.

One key element in the control of e-cigarette use in Taiwan will be effective regulation of the accessibility of e-cigarettes, particularly for young people. Although the government so far has continued to ban the importation of e-cigarettes according to the Pharmaceutical Affairs Law, the widespread availability and increasing prevalence of e-cigarette use attest to the insufficiency of such a passive control policy. More active regulation of the use of e-cigarettes could help to achieve better control of their booming use. In this regard, a recent proposal by the Health Promotion Administration in July 2017 for legislature to incorporate the regulation of e-cigarettes under the jurisdiction of the Tobacco Control Act points in the right direction. If passed, this amendment will allow the government to forbid the sale of e-cigarettes to people under the age of 18, enhance the deterrents to smuggling, and place a surcharge on e-cigarettes. The inclusion of a surcharge on e-cigarettes is particularly useful to curb the emerging use among young people, since this subpopulation is more sensitive to financial costs.^2^

Nevertheless, a purported benefit of e-cigarettes is that for certain populations, they provide one option for smoking cessation.^6^ However, our finding of a high cessation rate of e-cigarette use among dual users, which was also supported by previous studies,^40^^,^^54^ casts some doubt on this claim. Furthermore, the hazards of e-cigarettes have not been thoroughly examined.^7^^,^^55^ Taken together, our findings suggest that e-cigarettes should be regulated, and relevant products should be accurately labeled.

### Limitations

This study has some limitations. First, although more than 17,000 people participated in this national survey, the prevalence of e-cigarette use was low (ie, only 305 lifetime e-cigarettes users), especially exclusive e-cigarette users (27 people). Hence, the analysis of this group’s correlates is preliminary. Second, we did explicitly ask whether the c-cigarette cartridge consumed was nicotine containing or not. Third, the users of exclusive e-cigarettes were not required to complete the FTND. Nevertheless, this group of e-cigarette users reported using fewer cartridges than dual users, and their magnitude of nicotine dependence might be the lowest among the three groups of tobacco product users. Fourth, there were e-cigarette users whose initiation year was before the 2005 release date of modern e-cigarettes (40 out of 305 lifetime users, or 13%). We included these people in our analyses, except for in the analysis of the secular trend of e-cigarette initiation years. These e-cigarette users reported fewer cartridges consumed and were more likely to be dual users (82%) than those e-cigarette users whose initiation year was in 2005 or after. This potential misclassification error might weaken our estimates of the correlates with e-cigarette use. Fifth, the small number of adults with lifetime illicit drug use (148 people) render the relationship between e-cigarette use and illicit drug use preliminary. Finally, since this was a cross-sectional study, it did not allow us to infer causality on the relationships between tobacco use and various characteristics.
